# Hepatocellular carcinoma with gastric adenocarcinoma treated with atezolizumab and bevacizumab

**DOI:** 10.1002/ccr3.7875

**Published:** 2023-09-07

**Authors:** Takayoshi Suga, Yuko Kimura, Kensuke Furuya, Hiroko Sato

**Affiliations:** ^1^ Department of Gastroenterology Shibukawa Medical Center, National Hospital Organization Shibukawa Gunma Japan; ^2^ Department of Gastroenterology and Hepatology Gunma University Graduate School of Medicine Maebashi Gunma Japan

**Keywords:** double cancer, gastric cancer, immune checkpoint inhibitor, liver cancer, PD‐L1, VEGF

## Abstract

Atezolizumab and bevacizumab combination therapy might be one of the treatment options for hepatocellular carcinoma concurrent with gastric adenocarcinoma.

## Graphical abstract

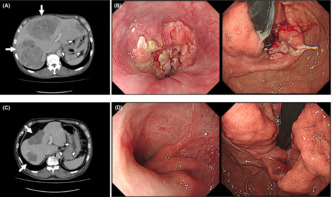

## 1 CASE

**FIGURE 1:**
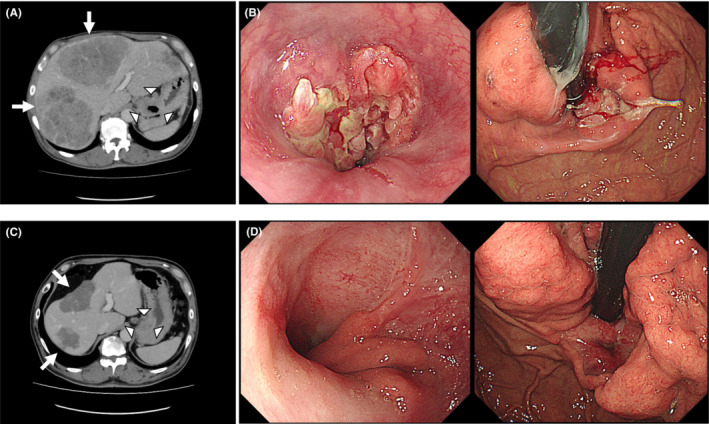
Computed tomography of hepatic and gastric masses before (A) and after (C) treatment. Endoscopy with gastric tumor before (B) and after (D) treatment.

A 58‐year‐old man was referred to our hospital for liver and gastric tumors. Abdominal contrast‐enhanced computed tomography (CT) revealed low‐density masses in the liver (Figure 1A, arrow) and stomach (Figure 1A, arrow head) with left supraclavicular lymph node metastasis. Upper gastrointestinal endoscopy revealed an ulcerous mass lesion in the gastric cardia (Figure 1B). The pathological diagnosis by liver biopsy was hepatocellular carcinoma and by gastrointestinal endoscopic biopsy was adenocarcinoma, showing programmed death‐ligand 1 (PD‐L1)‐positive and human epidermal growth factor receptor 2 (HER2)‐negative findings. Systemic chemotherapy for hepatocellular carcinoma was selected because the hepatic tumor was huge and thought to contribute to the patient's prognosis more than advanced gastric cancer. The patient was treated with atezolizumab and bevacizumab combination therapy, the first‐line treatment for hepatocellular carcinoma.^1^ Eighteen months after treatment, CT showed partial response of hepatocellular carcinoma (Figure 1C, arrow) and gastric adenocarcinoma (Figure 1C, arrow head). No tumor growth was observed on gastrointestinal endoscopy (Figure 1D).

To our knowledge, this is the first report that atezolizumab and bevacizumab combination therapy was effective in a patient with two primary cancers, namely, hepatocellular carcinoma and gastric adenocarcinoma. However, whether anti‐PD‐L1 antibody atezolizumab had any effect on gastric cancer in this case is controversial, and bevacizumab has been reported to have some effects.^2^, ^3^, ^4^ Therefore, selecting an effective treatment for two primary cancers is often difficult. Atezolizumab and bevacizumab combination therapy might be one of the treatment options for hepatocellular carcinoma concurrent with gastric adenocarcinoma.

## AUTHOR CONTRIBUTIONS

**Takayoshi Suga:** Writing – original draft. **Yuko Kimura:** Supervision. **Kensuke Furuya:** Supervision. **Hiroko Sato:** Supervision.

## CONFLICT OF INTEREST STATEMENT

There are no conflicts of interest to declare.

## CONSENT

Written informed consent was obtained from the patient to publish this report in accordance with the journal's patient consent policy.

## Data Availability

Data sharing not applicable to this article as no datasets were generated or analysed during the current study.
